# PTPN22 Acts in a Cell Intrinsic Manner to Restrict the Proliferation and Differentiation of T Cells Following Antibody Lymphodepletion

**DOI:** 10.3389/fimmu.2020.00052

**Published:** 2020-01-28

**Authors:** Johanna A. Knipper, David Wright, Andrew P. Cope, Bernard Malissen, Rose Zamoyska

**Affiliations:** ^1^Ashworth Laboratories, Institute of Immunology and Infection Research, University of Edinburgh, Edinburgh, United Kingdom; ^2^Faculty of Life Sciences and Medicine, Centre for Inflammation Biology and Cancer Immunology, School of Immunology and Microbial Sciences, King's College London, London, United Kingdom; ^3^Centre d'Immunologie de Marseille-Luminy, INSERM, CNRS, Aix Marseille Université, Marseille, France; ^4^Centre d'Immunophénomique, Aix Marseille Université, INSERM, CNRS UMR, Marseille, France

**Keywords:** lymphopenia, PTPN22, autoimmunity, regulatory T cells, interleukin-7

## Abstract

Lymphopenic insult has been shown to precipitate the initiation of autoimmune disease in murine models such as the Non-obese diabetic mouse. Similarly, in man lymphopenia induced by mAb therapy, for instance Alemtuzumab as treatment for Multiple Sclerosis, can precipitate development of secondary autoimmune disease in up to 30 % of patients. We asked whether an identified autoimmune susceptibility locus might increase the risk of developing autoimmunity in the context of mAb-induced lymphopenia in a mouse model. A single nucleotide polymorphism (SNP) in the gene encoding the tyrosine phosphatase PTPN22 (R620W) is associated with multiple human autoimmune diseases, and PTPN22 has been shown to modulate T cell responses, particularly to weak antigens. In keeping with this, PTPN22-deficient or PTPN22 R619W mutant murine T cells adoptively transferred into immunodeficient lymphopenic hosts showed a higher lymphopenia-induced proliferation rate than WT cells. We induced lymphopenia by treating wild-type or PTPN22 knock-out mice with T cell depleting antibodies and monitored reconstitution of the T cell pool. We found that PTPN22 deficient T cells acquired a more activated effector phenotype, with significantly more IFNγ producing cells. This resulted from expansion driven by self-peptide MHC, as it was evident when the contribution of IL-7 to lymphopenic expansion was blocked with IL-7R Ab. Interestingly, Foxp3^+^ Tregs were also considerably expanded in PTPN22-deficient and PTPN22 R619W mice, as was the frequency of both CD25^+^ and CD25^−^ CD4 T cells that produce IL-10. Using bone marrow chimeric mice, we showed that PTPN22 influenced development of both regulatory and effector T cell functions in a cell-intrinsic manner. Overall the expansion of Tregs is likely to keep the expanded T effector populations in check and sparing Treg during therapeutic mAb depletion may be a useful strategy to prevent occurrence of secondary autoimmunity.

## Introduction

Lymphopenia can lead to the expansion of autoreactive T cell clones and thus may be a risk factor for autoimmune diseases. Studies in mice have shown a direct link between lymphopenia-induced proliferation (LIP) and the development of diabetes in non-obese mice and autoimmune pancreatitis after sublethal irradiation and transfer of auto-antigen specific T cells (1, 2). However, in normal immune-competent mice lymphopenia induced T cell proliferation is well-tolerated with no signs of auto-inflammation (3). Thus, lymphopenia itself does not induce autoimmunity but has the potential, or can be a co-factor, in triggering autoimmune development by inducing alterations in the T cell repertoire and the balance between regulatory and conventional T cells. Initial rebalancing of homeostasis after a lymphopenic insult is mediated by cell proliferation, which is driven by recognition of MHC presented self-peptides via the T cell receptor and the greater availability of cytokines (4).

Lymphopenia is therapeutically induced for hematopoietic stem cell transplant (HSCT) therapies to treat a wide range of diseases (5, 6). Often HSCT therapies include lymphocyte depleting regiments, in order to condition for the subsequent transplant. Since T cell alloreactivity is a major risk for graft rejection, T cell depletion is an important preventive strategy in allogeneic HSCT although it comes with the cost of an increased risk of infection-mediated disease relapse and auto-inflammatory complications. However, so far only very limited data is available that investigates the risk of the development of autoimmune diseases post-conditioning and HSCT on a mechanistic level.

HSCT is also used to treat autoimmune diseases themselves, in which intense lymphodepleting condition regiments are used in order to target autoreactive cells. In a less invasive therapeutic approach to treat Multiple Sclerosis without undergoing irradiation, patients received alemtuzumab (Campath-1H), a monoclonal antibody against CD52. In this approach the immune system is reconstituted primarily by lymphopenia induced lymphocyte proliferation. Interestingly one third of the patients developed secondary autoimmune conditions after treatment with alemtuzumab, while another third presented with asymptomatic autoantibodies. Subsequently, Jones et al. showed that alemtuzumab-induced lymphopenia stimulated residual T cell expansion and a consequent shift in T cells toward a chronically activated phenotype with a loss of clonal diversity (7). Patients that developed a secondary autoimmunity showed a reduced TCR diversification compared to non-autoimmune patients. This study was the first to demonstrate a mechanistic link between lymphopenia induced proliferation and the development of autoreactive cells by characterizing T cell reconstitution in a group of lymphodepleted MS patients (7).

Notably, the risk of developing secondary autoimmunity following lympho-depletion regimes might be increased for these patients given that they started with an underlying autoimmune disease and therefore may have increased pre-disposition to self-reactivity. However, it is not understood why one patient develops secondary autoimmunity whereas another does not. Given that genome-wide association studies (GWAS) have identified numerous genetic mutations that act as risk factors for autoimmune disease, we asked whether genetic pre-disposition for autoimmunity increased the susceptibility to develop autoimmunity as a consequence of lymphopenia induced proliferation.

A single point mutation in the human *PTPN22* gene is associated with a higher risk for several autoimmune diseases such as rheumatoid arthritis, type 1 diabetes, systemic lupus erythematosus among others and is the strongest non-HLA risk factor described to date (8, 9). The *PTPN22* gene encodes a tyrosine phosphatase and is expressed in all hematopoietic cells. The risk variant *PTPN22*^*R*620*W*^ is relatively common in white Europeans and their descendants, with the highest incidence in Finland (15%) followed by Ukraine (14%) (10). The point mutation is located in the C-terminal domain of the PTPN22 protein, which is highly conserved between mice and men and functionally important for the interaction with CSK and TRAF3 (8). Studies in mice have demonstrated an important role for PTPN22 in negatively regulating T cell responsiveness to weak affinity antigens, resulting in an increased overall T cell number and an expansion of the memory-effector population in PTPN22 deficient mice (11, 12). A recent study showed that loss of PTPN22 in isogenic human Jurkat cells resulted in enhanced expression of IL-2 and CD69 upon antigen stimulation (13).

Given the relative high penetrance of the *PTPN22* variant allele, its association with autoimmunity and most importantly its role in regulating responses to weak affinity antigens, we investigated whether alterations in PTPN22 influenced the restoration of T cell homeostasis following perturbation *in vivo*. In keeping with clinical human lymphodepletion regimes, we induced severe lymphopenia by treating mice with monoclonal anti-CD4 and anti-CD8 depleting antibodies and monitored the reconstitution of the T cell pool. In this model upon reconstitution, PTPN22 deficient T cells acquired an enhanced activated effector phenotype, with significantly higher production of IFNγ compared to wildtype T cells. Interestingly we found that Foxp3^+^ Tregs are expanded and CD25^+^ as well as CD25^−^ CD4 T cells expressed higher levels of IL-10, which may keep the expanded effector population in check. Our data indicate that maintaining a high Treg:T effector cell ratio in mAb depletion therapies may be beneficial in preventing secondary autoimmunity.

## Materials and Methods

### Mice

Introduction of the R619W mutation was carried out by microinjection of a mix of Cas9 protein, guide RNA and ssDNA repair oligo (Integrated DNA Technologies) into single cell embryos generated by mating superovulated PEP-OST OT1 Rag1KO female mice, which contain a One-STrEP-Tag sequence knocked into the 5′ end of the PTPN22 gene (details below) with OT1 Rag1KO studs. Briefly, a 20 nt crRNA with sequence: AAGACTCGGGTGTCCGTTCA was designed to span the intended mutation site in exon 14 of PTPN22. This was annealed to TRACR RNA by heating to 95°C for 5 min and allowed to cool to room temperature before incubation with Cas9 protein at 37°C for 10 min in 0.1 TE buffer. The repair oligo was subsequently added to a final concentration of 100 ng/μL. The repair oligo was a 130bp ssDNA ultramer with sequence: CTTTATTCTTATAAAACATTGCGATTACATTTCTATTACTTGGTTAGCTCCTTCTCGGAGGACAGATGATGAAATCCCCCCGCCACTGCCTGAATGGACACCCGAGTCTTTTATTGTGGTTGAGGAAGCC which differed from the genomic DNA by three bases. The intended C to T change introduced a new XcmI restriction digest site and the arginine to tryptophan substitution. A further two silent mutations in the PAM region for the guide RNA were introduced to halt any subsequent Cas9 activity at this site. Introduction of the intended mutations were confirmed by restriction digest with XcmI and Sanger sequencing.

The PEP-OST OT1 Rag1KO mouse was generated by constructing a multitask allele of the *Ptpn22* gene comprising at its 5′ end a Twin-Strep-tag-coding sequence which encodes for the peptide tag OST (One-STrEP-Tag) and a Gly-Ser-Gly spacer sequence as well as two loxP sites bracketing the modified exon 1 (14). A frt-neo^r^-frt cassette was used for selecting ES cells containing the edited *Ptpn22* allele (15). M8.F6 C57BL/6N ES cells (16) were electroporated with the targeting vector. After selection in G418 or in G418 plus ganciclovir, ES cell clones were screened for proper homologous recombination by Southern blot and PCR analysis. A probe specific for the neo^r^ cassette was also used to ensure that adventitious non-homologous recombination events had not occurred in the selected clones. Mutant ES cells were injected into FVB blastocysts. Excision of the frt-neo^r^-frt cassette was achieved through cross with transgenic mice expressing a FLP recombinase under the control of the actin promoter (15). Screening for the presence of the OST-targeted alleles was performed by PCR using the following pair of primers: sense 5′-GCAGTGGCTTCTTGGTGCTG-3′ and antisense 5′-TGGCAAACTCCTCACTGTTG-3′. The official name given to those *Ptpn22*^OST^ mice is B6-*Ptpn22*^tm2Ciphe^. Mice carrying the PEP-OST modification were subsequently back-crossed onto the Rag1KO OT-I background.

Mice with a global PTPN22 deficiency, were backcrossed >10 times to the C57BL/6J background and crossed to a Rag1KO OT-I background as previously described (12). Rag1 KO, C57BL6/J, CD45.1, CD45.1/2, and CD45.2 mice were bred in-house at the University of Edinburgh. Age-matched (7–12 weeks at start of experiment) and sex-matched mice were used in all experiments. Untreated control mice were co-housed with experimental mice and had a similar age at necropsy. All procedures were approved under a project license granted by the UK Home Office and performed in accordance with the institutional and ethical guidelines of the University of Edinburgh.

### Antibody-Mediated T Cell Lymphopenia

To induce severe T cell lymphopenia 100 μg anti CD4 (clone YTS 191) and 50 μg of anti CD8 (clone YTS 169.4), both BioXcell were given intraperitoneally. Where stated, groups of mice were given 300 μg mAb to IL-7R (clone A7R34; a hybridoma prepared in-house) intraperitoneally every second day over the 14 days experimental time-course. The anti CD25 antibody (clone PC61) was purchased from BioXcell and a single dose of 500 μg per mouse was injected intraperitoneally.

### Adoptive T Cell Transfer Experiments

For cell transfer into Rag-1 KO recipient mice, CD45.1 PTPN22 WT, CD45.1/2 PTPN22 R169W, and CD45.2 PTPN22 KO OT-I T cells were mixed at a ratio of 1:1:1 and a total of 5x10e5 cells were injected intravenously. In some experiments, recipient mice were lethally irradiated before cell transfer. Mixed bone marrow chimeras were generated by reconstituting CD45.1/2 PTPN22 WT recipients with T cell depleted bone marrow cells from CD45.1 PTPN22 WT and CD45.2 PTPN22 KO mice, mixed at a ratio of 1:1. In other experiments CD45.1/2 recipients were reconstituted with Rag1 KO bone marrow and received in addition 2.5x10e5 CD45.1 WT and CD45.2 PTPN22 KO naïve CD4^+^CD62L^+^CD44^−^CD25^−^ T cells mixed at a ratio of 1:1. Naïve CD4 T cells were obtained by enrichment of CD4^+^ T cells using a mouse CD4^+^ T cell Isolation Kit (Miltenyi), followed by sorting for naive CD4^+^CD62L^+^CD44^−^CD25^−^ T cells with a FACSAria sorter (BD).

### Flow Cytometry

The following specific antibodies were used: a(anti)CD4 fluorescin isothiocyanate (Fitc), allophycocyanin (APC) or Pacific Blue (clone RM4-5), aCD4 phycoerythrin (Pe) cyanine (Cy)7 or Pacific Blue (clone RM4-4), aCD8 Fitc or peridinin chlorophyll protein (PerCP)/Cy5.5 (clone YTS156.7.7), aCD25 PeCy7 (clone 3C7), aCD45 PerCP/Cy5.5 (clone 30-F11), aCD45.1 Fitc or APC (clone A20), aCD45.2 PcP, PeCy7 or Pacific Blue (clone 104), aCD62L Pe, APC or Pacific Blue (clone MEL-14), aIFNγ Fitc, PeCy7 or APC (clone XMG1.2), aIL-10 Pe or APC (clone JES5-16E3) and aIL-10R (aCD210) Pe (clone 1B1.3a) were purchased from BioLegend. aCD8 PeCy7 (clone eBioH35-17.2), aCD25-APC or PeCy7 (clone PC61.5), aCD44 Alexa eFlour780 (clone IM7), aFoxp3 eFlour 450, Pe or APC (clone FJK-16s) and aT-bet Pe (clone 4B10) were purchased from eBiosciences. aKi67-PE or Fitc (clone B56) and aCD4-Fitc (RM4-5) were purchased from BD. Live/Dead Aqua or Far Red (both Life Technologies) were used to identify live cells. Intracellular cytokine staining was performed using Fixation buffer (BioLegend) to permeabilise and fix cells, for subsequent antibody staining the Intracellular Perm Wash Buffer (BioLegend) was used. For staining of transcription factors, cells were permeabilized and fixed in FoxP3 Fix/Permeabilization Buffer (eBioscience) before staining with antibodies in Permeabilization/Wash buffer (eBioscience). Samples were acquired with a Fortessa (BD) or a MACSQuant flow cytometer (Miltenyi). Data was analyzed with FlowJo software (Treestar).

### Cell Culture and Stimulation

Cells from lymph nodes or spleens were cultured in IMDM medium supplemented with heat-inactivated 10% FBS, 2 mM L-glutamine, 100 U/mL Penicillin, 100 mg/mL Streptomycin and 50 μM 2-mercaptoethanol (all Sigma-Aldrich). For *in vitro* cell proliferation assays cells were labeled with 500 nM CellTracer Violet (Life Technologies). N4, T4, and G4 peptides (Peptide Synthetics) were added to culture medium at indicated concentrations. Recombinant IL-7 (Peprotech) was used at 20 ng/mL, recombinant IL-10 (Peprotech) was used at indicated concentrations. For cytokine recall responses cells were restimulated with 20 ng/mL phorbol 12,13-dibutyrate (PdBU) and 0.5 μg/mL Ionomycin (all Sigma-Aldrich) in presence of 1 μg/ml Brefeldin A for 4 h before intracellular staining (antibodies identified above). Transgenic OT-1 T cells were restimulated with T4 peptide (SIITFEKL) in presence of 1 μg/ml brefeldin A for 4 h. Where stated cells were stimulated in media with 0.5 μg/mL anti CD3ε antibody and 2 μg/mL anti CD28 antibody (both BioLegend) for 4 h.

### Statistical Analysis

Prism software Version 7 was used for statistical significance analysis. All data was tested for normality and depending on the experimental set up a Student's *t*-test or a 1way ANOVA with a Sidak's multiple comparison test was performed. For experiments with two factors a 2way ANOVA test was used followed by Sidak's multiple comparison test. In experiments in which cells of different genotypes were adoptively transferred into one host a paired analysis was used. When a dataset failed to meet the requirements for a parametric test, a non-parametric unpaired Kruskal Wallis test with a Dunn's multiple comparison test was used. All experiments were repeated at least twice. The figure legends are stating the number of repeats for each experiment and how many animals were used.

## Results

### Similar T Cell Reconstitution in WT and *Ptpn22* KO Mice Following Transient Lymphopenia

**Figure 1:**
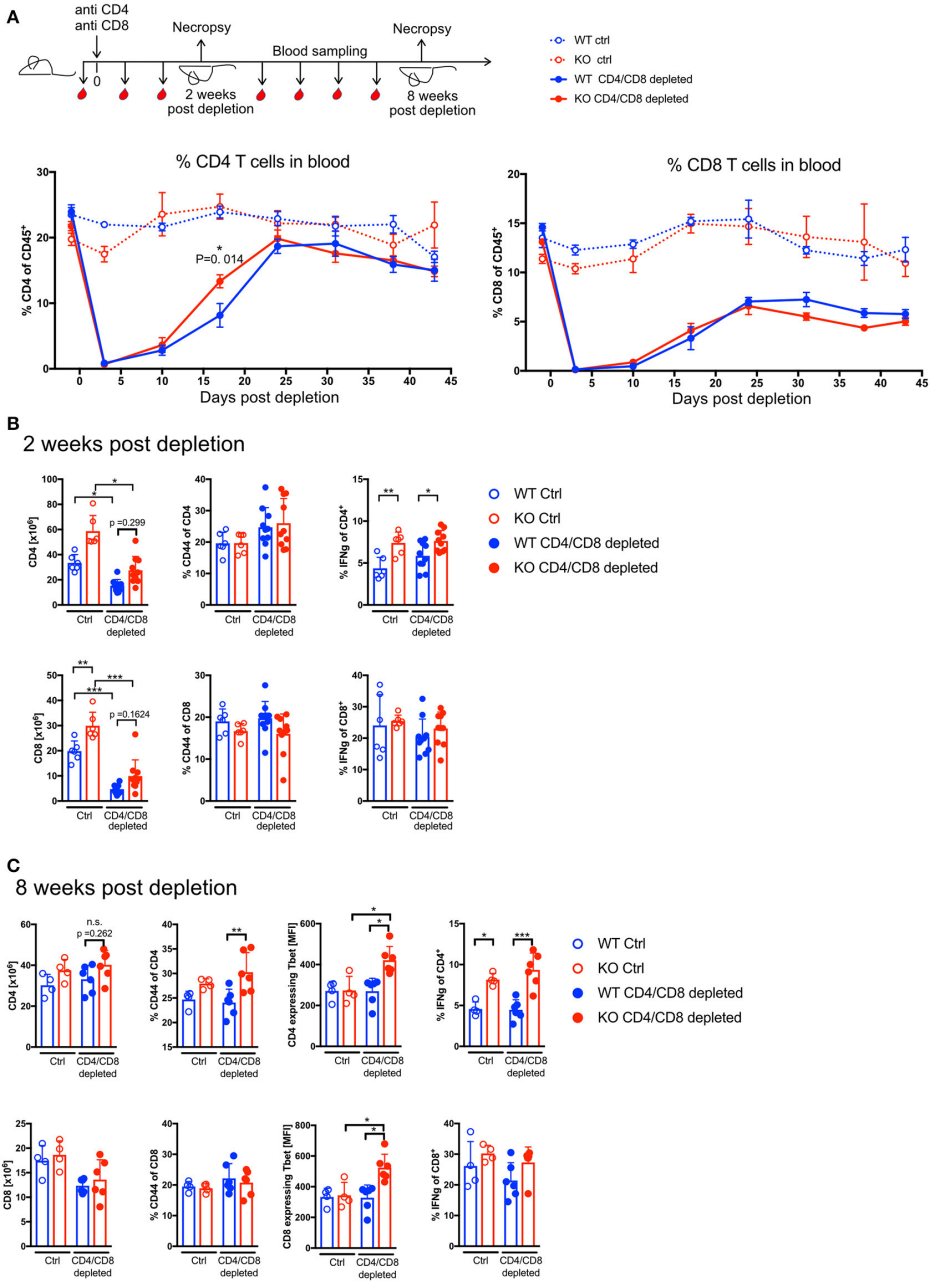
PTPN22 controls T cell effector phenotype but does not affect T cell proliferation rate in response Ab-mediated lymphopenia. **(A)** Scheme of experimental set-up. T cell expansion was assessed by measuring CD4 and CD8 T cell frequencies in blood before T cell depletion throughout the length of the experiment. Two-way ANOVA with Sidak's multiple comparison test. *N* = 4–6 animals per group, one representative experiment out of three independent experiments in total. Absolute T cell numbers and T cell phenotype analysis in Spleen 2 weeks post depletion **(B)** and 8 weeks post depletion **(C)**. Each dot represents one animal, data pooled of two independent experiments in **(B)** with *N* = 6–10 animals per group. Data in **(A,C)** shows one representative experiment out of three independent experiments in total with *N* = 4–6 animals per group. Two-way ANOVA with Sidak's multiple comparison test. n.s. = non-significant. A *p* < 0.05 was considered significant. ^*^p < 0.05, ^**^p < 0.01, ^***^p < 0.001.

To test whether *Ptpn22* regulated homeostatic T cell proliferation in response to transient lymphopenia in mice we chose an Ab-mediated lymphopenia model, as this was similar to the mode of action of drugs such as Alemtuzumab which is used for lymphodepletion therapy in man. A single injection of 100 μg CD4 and 50 μg CD8 mAbs per mouse mediates severe T cell depletion with high specificity (3). At day 3 post injection 90–99% of CD4 and CD8 T cells were depleted in blood, spleen and lymph nodes (Supplementary Figures 1A,B). The depletion efficacy was similar in *Ptpn22* wildtype (WT) and *Ptpn22* knockout (KO) mice (Supplementary Figures 1A,B). After depletion of T cells, lymphocyte reconstitution was monitored by blood sampling. The absence of PTPN22 had very little effect on the overall kinetics of T cell reconstitution and both PTPN22 WT and KO CD4 T cells reached homeostasis around 4 weeks after depletion (Figure 1A). In contrast, proportions of CD8 T cells were not fully restored to pre-treatment frequencies during the course of the experiment. The frequency of bulk CD4 and CD8 T cells in blood increased steadily over time with no overall significant difference between PTPN22 WT and KO mice (Figure 1A). CD4 T cells showed a transiently higher frequency in PTPN22 KO blood around 2 weeks post-treatment (Figure 1A), and were slightly elevated in PTPN22 KO spleen, although this difference did not reach significance (>*p* = 0.05) (Figure 1B), suggesting that PTPN22 has a minor impact on the proliferation rate.

Phenotypically T cells acquire an effector memory phenotype during proliferation in response to homeostatic cues (4). Two weeks post depletion the frequency of CD44^+^ cells was slightly elevated in CD4 T cells in comparison to untreated mice where there was no difference between the genotypes. However, IFNγ expression was significantly increased in PTPN22 KO CD4 T cells indicating that PTPN22 influences the inflammatory potential of T helper cells and the difference between WT and KO was particularly pronounced in T cells of mice that underwent lymphopenia induced proliferation (Figure 1B). Eight weeks post depletion CD4 T cell numbers were comparable to untreated control mice, whereas CD8 T cell counts are still lower in the depleted groups compared to steady state (Figure 1C).

Phenotypic analysis of the T cells showed increased frequency of CD44^+^ and significantly higher expression of Tbet and IFNγ in PTPN22 KO CD4 T cells after they underwent lymphopenia induced proliferation. Similarly, PTPN22 KO CD8 T cells had significantly more Tbet positive cells following reconstitution but their proportions of CD44^+^ and IFNγ^+^ cells were not significantly increased. Together these data show that many weeks after restoration of homeostasis, PTPN22 deficient T cells have a more activated phenotype than WT cells (Figure 1C). Whilst activated T cells have the potential to cause (auto-) inflammation, even after a very long period of more than half a year post-depletion, PTPN22 KO mice did not develop any overt signs of autoaggression as determined by signs of weight loss, indicating inflammatory colitis, a response known to be exacerbated by loss of PTPN22 following lymphopenic expansion after transfer of naive T cells into lymphopenic hosts (17) or other overt post-mortem signs of organ damage (data not shown).

### PTPN22-Regulated T Cell Expansion Is Independent of IL-7Rα Signaling

**Figure 2:**
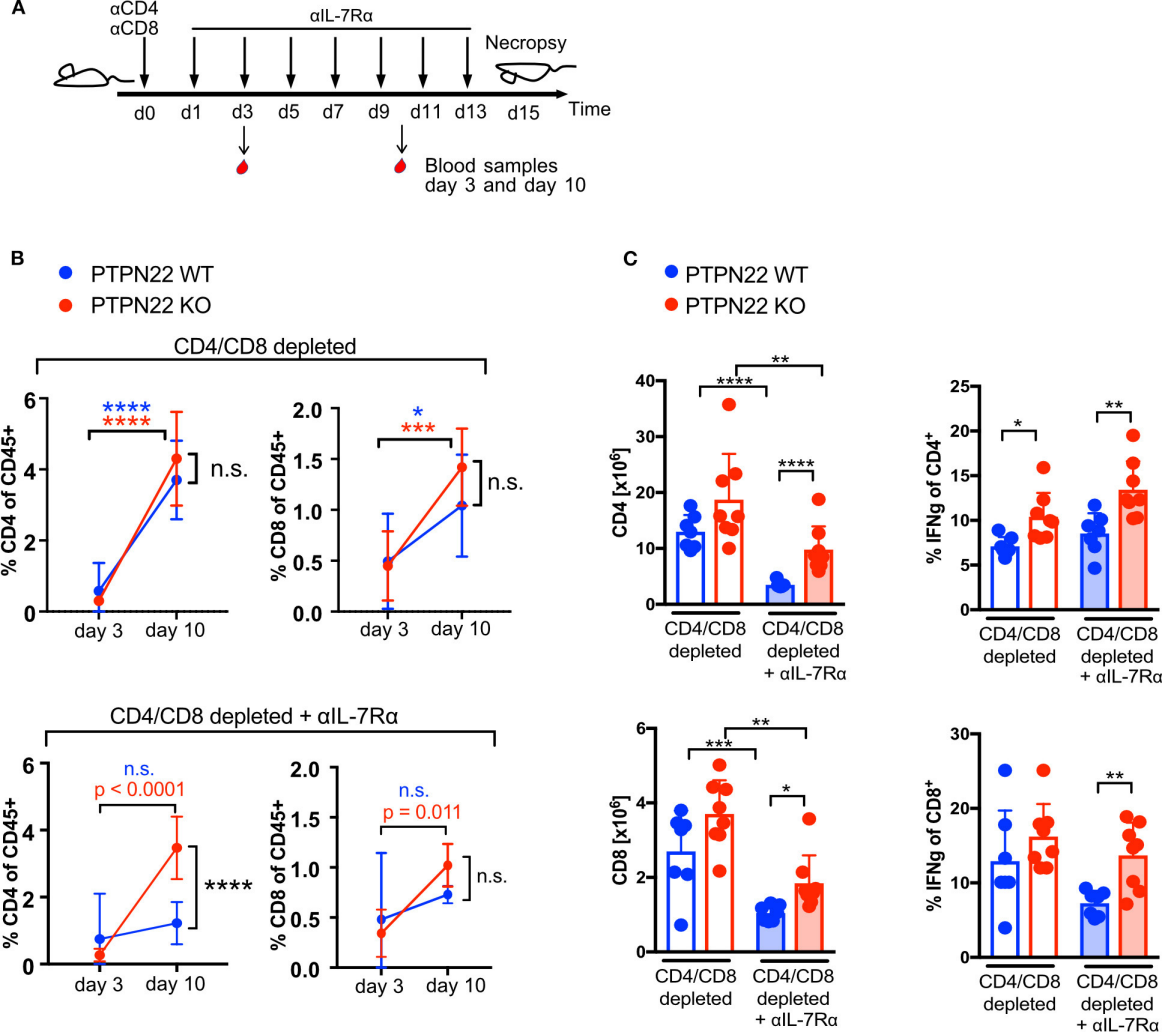
PTPN22 controls T cell proliferation when IL-7 availability is limited. **(A)** Scheme of experimental set-up. **(B)** CD4 and CD8 T cell frequency in blood 3 and 10 days post T cell depletion in combination with IL-7Rα blockade. Data means with SD shown, *N* = 7 animals per genotype and time point. Two-way ANOVA with Sidak's multiple comparison test. **(C)** Analysis of T cells number and IFNγ expression in spleen 2 weeks post T cell depletion or T cell depletion in combination with IL-7 Rα blockade. Each dot represents one animal, *N* = 7–8 animals per group, shown data is pooled of two independent experiments. Two-way ANOVA with Sidak's multiple comparison test. A *p* < 0.05 was considered significant ^*^p < 0.05, ^**^p < 0.01, ^***^p < 0.001, ^****^p < 0.0001.

IL-7 signaling contributes to T cell proliferation in response to lymphopenia and can mask TCR mediated proliferation (18). To understand the contribution of PTPN22 in TCR mediated homeostatic proliferation following Ab depletion, IL-7 signaling was inhibited by the injection of an IL-7 receptor α blocking antibody (IL-7Rα Ab) every other day (Figure 2A). Blood sampling showed that T cells increased in frequency from day 3 to 10 after T cell depletion in both WT and KO mice, with CD4 T cells increasing 6 to 14-fold and CD8 T cells 1.5 to 3-fold (Figure 2B, top panel). Addition of IL-7Rα Ab markedly reduced proliferation in T cells compared to control mice receiving no IL-7Rα Ab, such that WT cells increased in proportion by only 1.6 times for CD4 and 1.5 times for CD8 between day 3 and 10. In contrast, even in the presence of IL-7Rα Ab, PTPN22 KO mice showed a significant increase of CD4 (12.9-fold) and CD8 T cells (3.0-fold) in blood in the same time span (Figures 2B,C). Analysis of the spleen confirmed significantly higher CD4 and CD8 cell numbers in PTPN22 KO compared to WT mice when IL-7R signaling was blocked together with an increase in IFNγ producing CD4 and CD8 T cells (Figures 2B,C). Thus, blockade of IL-7Rα signaling suggests that PTPN22 KO cells proliferated faster to TCR-mediated lymphopenic expansion, resulting in overall higher T cell numbers and an increased capacity to produce IFNγ 2 weeks after Ab depletion.

### Foxp3+ Tregs Are More Resistant to Ab-Mediated T Cell Depletion

**Figure 3:**
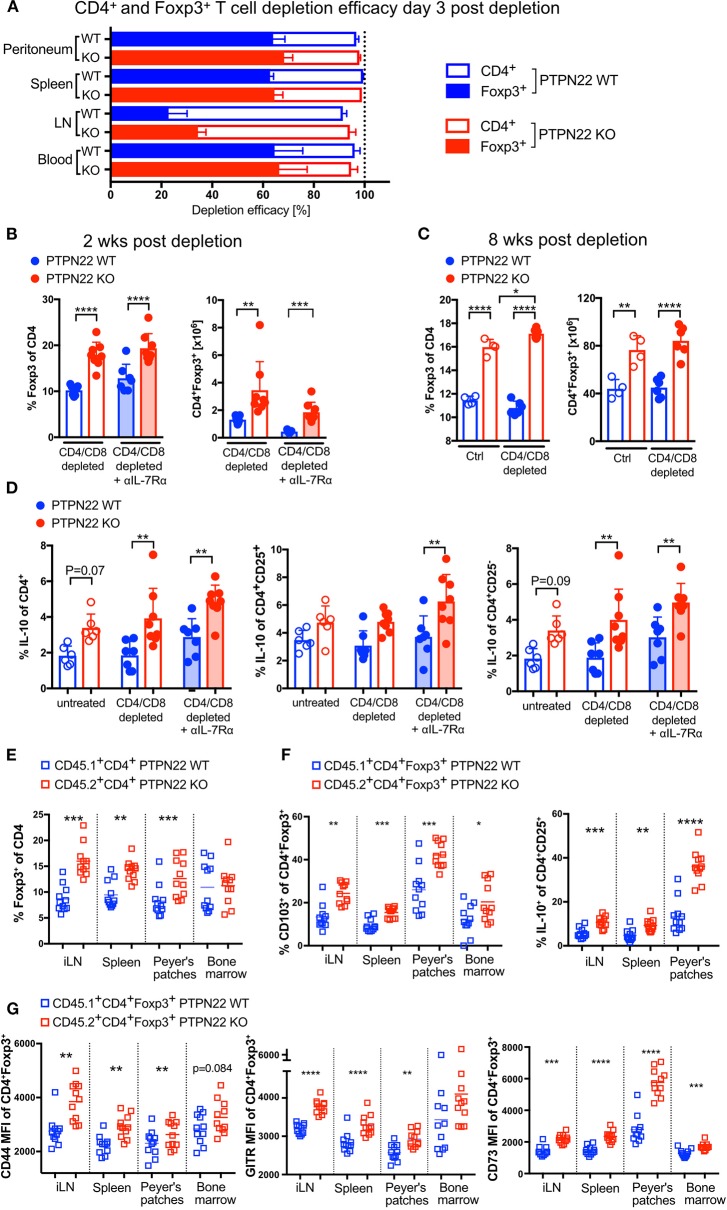
Foxp3^+^ Tregs and IL-10 production are cell-intrinsically controlled by PTPN22 in steady state and LIP. **(A)** Ab-mediated depletion efficacy of CD4^+^Foxp3^+^ Tregs and CD4^+^Foxp3^−^ conventional CD4 T cells 3 days post anti-CD4 and anti-CD8 injection. Data shown as mean with SD with *N* = 7 animals per group. Pooled data of two independent experiments. **(B)** Proportion and absolute numbers of regulatory T cells 2 weeks after T cell depletion in combination with IL-7R blockade and **(C)** 8 weeks post depletion. Each dot represents one animal. In **(B)** *N* = 7–8 animals per group, shown data is pooled of two independent experiments. In **(C)** *N* = 4–6 animals per group, one representative experiment out of three independent experiments. Two-way ANOVA with Sidak's multiple comparison test. **(D)** IL-10 production by CD4 T cells, CD4^+^CD25^+^ Tregs and CD4^+^CD25^−^ conventional T cells 2 weeks post depletion. To analyze IL-10 production cells were re-stimulated in presence of Brefeldin A *ex vivo*. Each dot represents one animal. Each dot represents one animal. *N* = 6–8 animals per group, shown data is pooled of two independent experiments. Two-way ANOVA with Sidak's multiple comparison test. **(E)** Bone marrow from CD45.1 PTPN22 WT and CD45.2 PTPN22 KO donor animals was mixed 1:1 and transplanted in into fully irradiated CD45.1/2 PTPN22 WT recipient mice. The Foxp3^+^ frequency within PTPN22 WT CD45.1^+^CD4^+^ or PTPN22 KO CD45.2^+^CD4^+^ cell pool was assessed 15 weeks post transfer. Each dot represents one animal (*N* = 11), data is pooled of two independent experiments. A paired *t*-test was performed to compare PTPN22 WT and KO cells within the same recipient. **(F,G)** Phenotypical analysis of Foxp3^+^ expressing PTPN22 WT CD45.1^+^CD4^+^ and PTPN22 KO CD45.2^+^CD4^+^ cells from the experiment outlined in **(E)**. Each dot represents one animal (*N* = 11), data is pooled of two independent experiments. A paired *t*-test was performed to compare PTPN22 WT and KO cells within the same recipient. A *p* < 0.05 was considered significant ^*^p < 0.05, ^**^p < 0.01, ^***^p < 0.001, ^****^p < 0.0001.

Regulatory T cells are known to suppress T cell proliferation and activation and are likely to be involved in regulating lymphopenia-induced proliferation following Ab depletion. Analysis of different sites and organs showed that in contrast to the highly efficient depletion of CD4 T cells, only between 20 (lymph nodes) and 60% (spleen, blood, peritoneum) of Tregs were depleted 3 days after antibody injection (Figures 3A, Supplementary Figure 1C); the overall depletion efficacy in PTPN22 WT and KO mice was similar. This data suggested that Ab-mediated CD4 depletion spares a large proportion of Tregs.

PTPN22 KO animals have increased numbers of Foxp3^+^ Treg cells in steady state (17, 19) and 2 weeks post-depletion PTPN22 KOs already showed a significantly higher proportion of Foxp3 expressing cells in comparison to WT mice, independently of IL-7 availability (Figure 3B). Eight weeks post-depletion, at which point CD4 homeostasis has been restored, there were significantly higher numbers of Foxp3^+^ T cells in PTPN22 KO mice, similar to steady state untreated control mice (Figure 3C). In addition, a higher frequency of PTPN22 KO CD4 cells produced IL-10, and these comprised both CD25^+^ and CD25^−^ CD4 T cells (Figure 3D).

In order to address whether the increased frequency of Treg in reconstituting PTPN22 KO mice was influenced by the presence of other PTPN22 KO hematopoietic cells (e.g., dendritic cells or macrophages), lethally irradiated CD45.1/2 PTPN22 WT mice were reconstituted with mixed BM from CD45.1 PTPN22 WT and CD45.2 PTPN22 KO mice in a 1:1 ratio. In this scenario, newly formed T cells exit the thymus into a lymphopenic adult host and undergo homeostatic expansion and we should expect to observe similarly expanded Treg populations in both WT and PTPN22 KO T cells if this is driven by the presence of PTPN22 KO hematopoietic cells. Fifteen weeks after BM transplant we found higher Foxp3^+^ frequencies within the PTPN22 KO CD4 pool compared to the corresponding WT CD4 pool from each individual mouse. These data indicate that Treg expansion is intrinsically regulated by the presence of PTPN22, rather than being driven by cytokine production from other PTPN22 KO hematopoietic cells, which would have similarly affected both WT and KO Treg generation (Figure 3E). Further analysis revealed that PTPN22 KO Foxp3^+^ express higher levels of CD44 and PTPN22 KO CD25^+^ produced more IL-10 in response to re-stimulation, and have a more activated phenotype (CD44+, CD103+, GITR+ and CD73+) than the co-generated WT Treg, indicating that their acquisition of regulatory function and state of activation is also regulated by PTPN22 (Figures 3F,G).

We hypothesized that the expanded Foxp3^+^ CD4 T cells may be restraining lymphopenia-induced proliferation in the context of the PTPN22 KO and attempted to address this point by specific depletion of Treg using an anti-CD25 antibody, but were unsuccessful due to the poor depletion efficacy of this antibody (Supplementary Figure 2). As an alternative approach we performed co-transfers of naïve T cells into irradiated recipients to follow the expansion of PTPN22 KO and WT cells in the absence of Treg.

### PTPN22 KO T Cells Undergo More Lymphopenic Expansion Than WT T Cells in the Absence of Tregs

**Figure 4:**
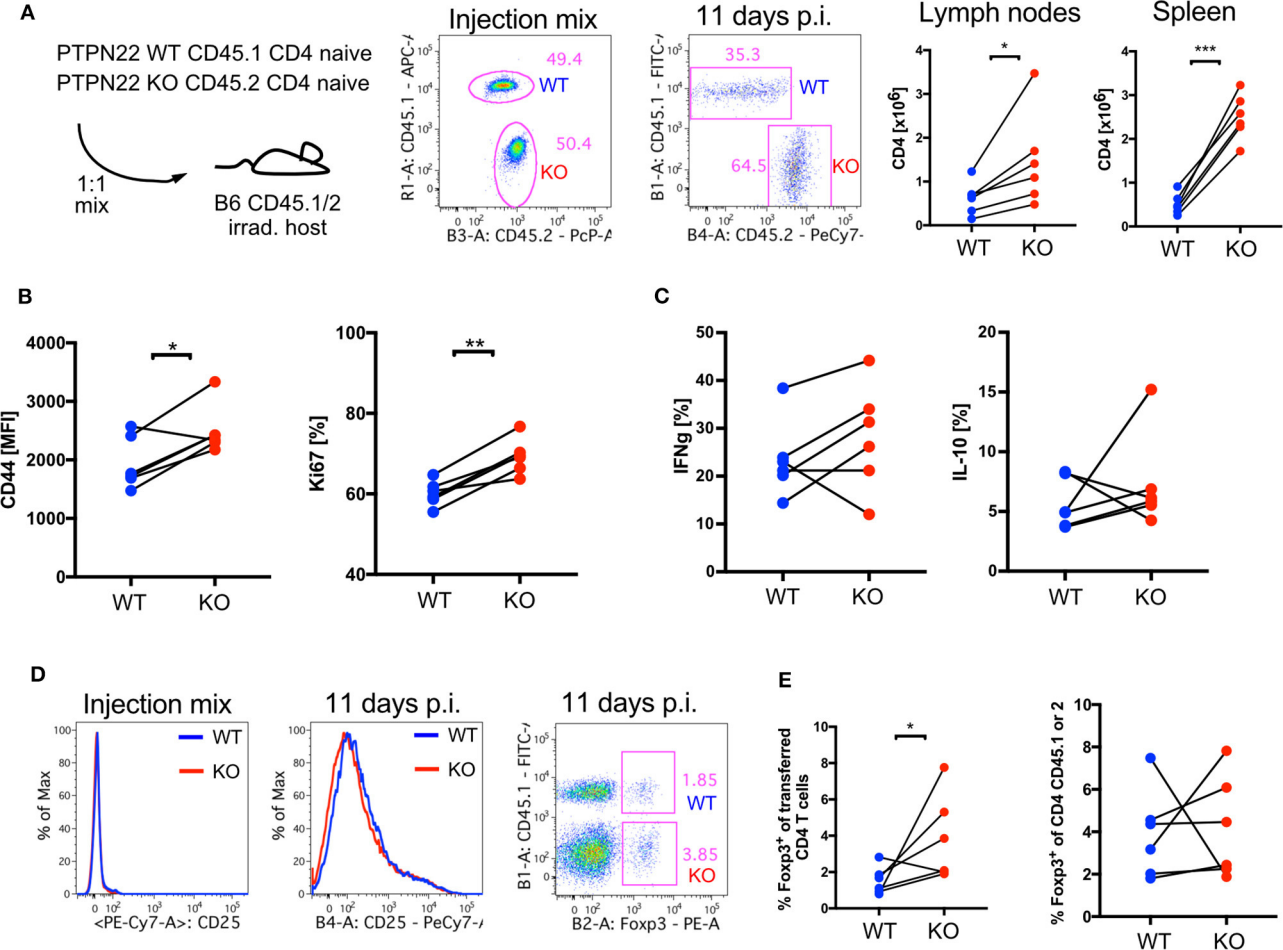
CD4 PTPN22 KO cells outnumber WT when co-transferred into lymphopenic host. **(A)** Adoptive transfer of naive CD4^+^CD45.1^+^ PTPN22 WT and CD4^+^CD45.2^+^ PTPN22 KO cells into lethally irradiated CD45.1/2 WT hosts in a 1:1 ratio. Ratio of transferred cells was assessed 11 days post transfer in spleen and lymph nodes. **(B)** Analysis of transferred cells 11 days post transfer for expression of CD44, Ki67 and **(C)** cytokines IL-10 and IFNγ. **(D)** Expression of CD25 before transfer and expression of Foxp3 of transferred CD4 T cell population post transfer. **(E)** Frequency of Foxp3^+^ expressing cells within the CD4^+^CD45.1^+^ PTPN22 WT cell population in comparison to Foxp3 expressing cells within the CD4^+^CD45.2^+^ PTPN22 KO cell population. Data shown in **(A–E)** is from one representative experiment of two independent experiments is shown with *N* = 6 animals. Each dot represents one animal. CD45.1^+^ PTPN22 WT and CD45.2^+^ PTPN22 KO cells obtained from the same host were compared by a paired *t*-test in **(A–E)**. A *p* < 0.05 was considered significant ^*^p < 0.05, ^**^p < 0.01, ^***^p < 0.001.

PTPN22 is expressed in all hematopoietic cells and regulates production of cytokines which could affect peripheral T cell expansion in response to lymphopenia in the Ab depletion models we were analyzing. In order to further examine whether either non-T cells and/or Treg influenced the proliferation of PTPN22 KO T cells in lymphopenic animals, naïve CD4^+^CD62L^+^CD44^−^CD25^−^ PTPN22 WT and KO CD4 T cells were transferred into lethally irradiated CD45 congenic C57Bl/6 WT hosts which had been treated with antibiotics to prevent the fast proliferation stimulated by gut antigens (4). Eleven days after transferring 2.5 x 10^5^ PTPN22 WT and KO cells at a ratio of 1:1 there was a change in the ratio of WT:KO to 1:2. Higher numbers of PTPN22 KO compared to WT CD4 T cells were recovered from lymph nodes and spleen, further confirming that the influence of PTPN22 on lymphopenia induced T cell proliferation was cell intrinsic and independent of the presence of other PTPN22 KO hematopoietic cells (Figure 4A). PTPN22 KO CD4 T cells also had a higher abundance of CD44 expression and a larger proportion expressed Ki67, a marker for cell proliferation (Figure 4B), whereas at this time point expression of IFNγ and IL-10 was comparable between WT and KO cells (Figure 4C).

The transferred T cells were sorted for naïve T cells, so lacked Treg, but after 11 days a proportion of the input cells became CD25^+^. Further analysis showed that up to 11% of the cells expressed Foxp3 so became iTreg, with significantly higher contribution from the PTPN22 KO cells to the overall Foxp3 positive population (Figure 4D). Importantly, equivalent frequencies of Tregs were found within gated WT or KO CD4 populations, indicating that the increased absolute number of Foxp3^+^ PTPN22 KO Treg was a consequence of the overall higher abundance of PTPN22 KO CD4 T cells within the population of recovered cells (Figure 4E). These data indicate that the rate of differentiation of naïve T cells into regulatory cells was not affected by the presence of PTPN22.

### PTPN22^R619W^ T Cells Recapitulate the Response of PTPN22 KO Cells to Lymphopenia

**Figure 5:**
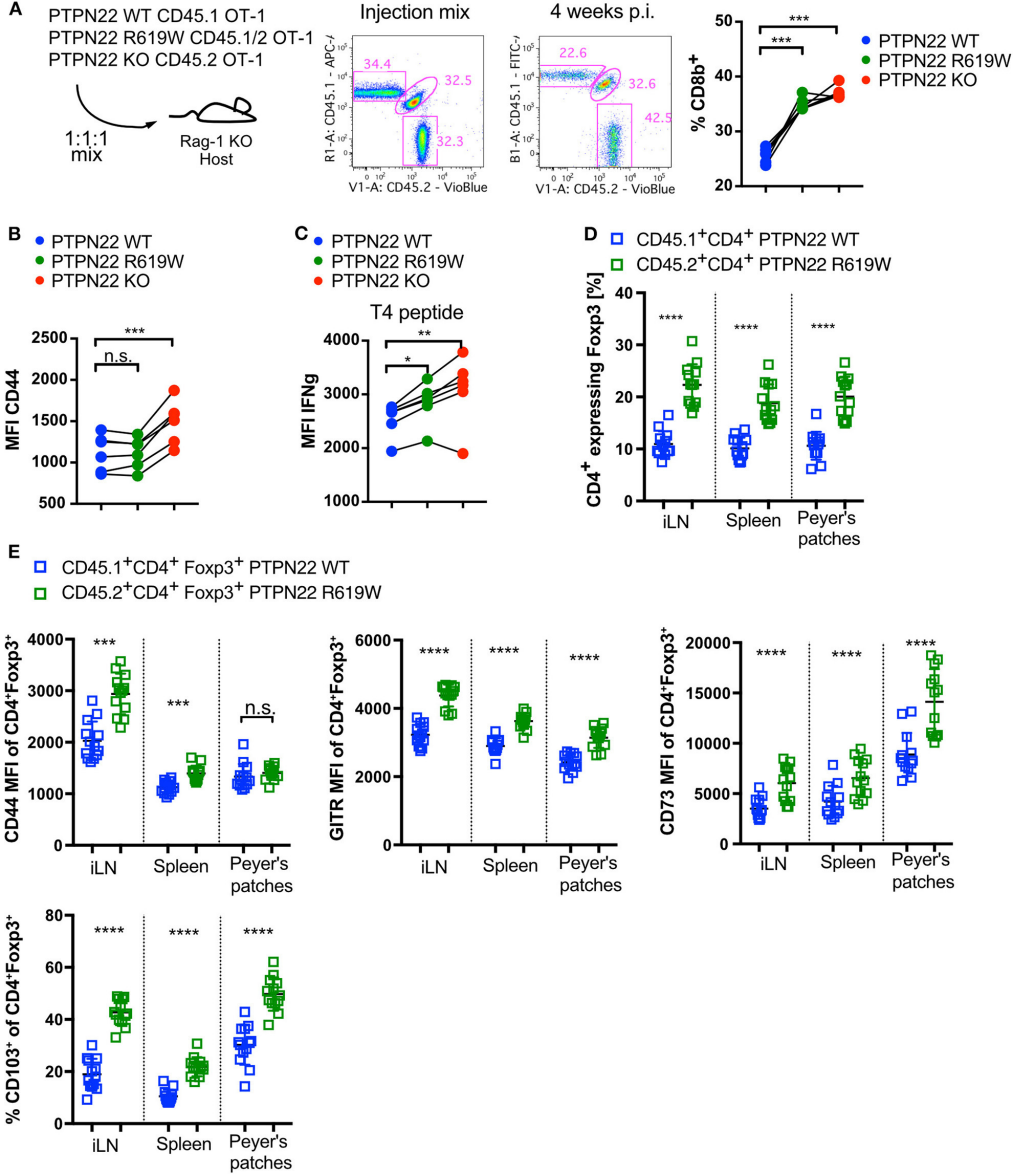
PTPN22 R619W negatively regulates T cell proliferation in response to lymphopenia. **(A)** Adoptive transfer of CD45.1 PTPN22 WT, CD45.1/2 PTPN22 R619W and CD45.2 PTPN22 KO CD8 OT-1 cells in a 1:1:1 ratio into lymphopenic Rag-1 KO hosts. Frequency of CD8 T cells from the different genotypes were analyzed 4 weeks after transfer. **(B)** Analysis of CD44 expression and **(C)** IFNg expression in response to re-stimulation with T4 peptide 4 weeks post transfer. Data shown in **(A–C)** is from one representative experiment out of two independent experiments with *N* = 5 recipient animals, each dot represents one animal. PTPN22 WT, R619W and KO cells were compared by paired one-way ANOVA. **(D)** CD45.1 PTPN22 WT and CD45.2 PTPN22 R619W bone marrow was mixed 1:1 and transplanted in into fully irradiated CD45.2 PTPN22 R619W recipient mice. The Foxp3^+^ frequency within PTPN22 WT CD45.1^+^CD4^+^ or PTPN22 R169W CD45.2^+^CD4^+^ cell pool was assessed 15 weeks post transfer. Each dot represents one animal (*N* = 13), data is pooled of two independent experiments. A paired *t*-test was performed to compare PTPN22 WT and R619W cells within the same recipient. **(E)** Phenotypical analysis of Foxp3^+^ expressing PTPN22 WT CD45.1^+^CD4^+^ and PTPN22 KO CD45.2^+^CD4^+^ cells from the experiment outlined in **(D)**. Each dot represents one animal (*N* = 13), data is pooled of two independent experiments. A paired *t*-test was performed to compare PTPN22 WT and R619W cells within the same recipient. n.s., non-significant. A *p* < 0.05 was considered significant ^*^p < 0.05, ^**^p < 0.01, ^***^p < 0.001, ^****^p < 0.0001.

The previous data suggested that PTPN22 acts in a cell intrinsic manner to restrict the proliferation and differentiation of WT T cells in a lymphopenic environment. As the PTPN22^R620W^ variant is found in man rather than a full KO, we tested whether the equivalent mutation in mouse (R619W) behaved more similarly to either PTPN22 WT or KO cells in the context of lymphopenia. First, in order to exclude extrinsic influences of other hematopoietic populations and in particular Treg on lymphopenia-induced expansion, we used TCR transgenic OT-1 Rag-1 KO CD8 T cells, and adoptively transferred these cells into Rag-1 KO lymphopenic hosts. The availability of transgenic OT-1 CD8 T cells expressing CD45 congenic markers on the three genotypes, PTPN22 WT, KO, and R619W, allowed us to compare all genotypes in the same environment. The use of transgenic OT-1 CD8 cells, which express a T cell receptor that recognizes chicken ovalbumin, precludes responses to bacterial components from gut which can drive a much faster homeostatic expansion than the slow proliferation seen in response to self-peptide:MHC (4). CD8 cells expanded in Rag-1 KO hosts and 4 weeks after transfer, the initially equal cell ratio shifted clearly in favor of PTPN22 R619W and PTPN22 KO cells (Figure 5A). PTPN22 KO CD8 cells showed higher expression of CD44 than PTPN22 WT or PTPN22^R619W^ cells (Figure 5B). When cells recovered from the lymphopenic mice were re-stimulated with T4, a low affinity ligand for the OT-1 T cell receptor, IFNγ production was higher in PTPN22 KO compared to WT OT-1 cells, with PTPN22^R619W^ showing an intermediate phenotype in comparison to the full knock out (Figure 5C).

We asked whether the tendency to generation of more Treg was also evident in the R619W mice by making mixed bone marrow chimeras between R619W and WT bone marrow using CD45 allelic markers to distinguish them. Similar to the PTPN22 KO/WT mixed chimeric mice, increased numbers of Tregs with a more activated phenotype (CD44+, CD103+, GITR+, CD73+) were recovered with the R619W compared with the WT genotype (Figures 5D,E).

Together these data show that proliferation induced by lymphopenia is intrinsically regulated by PTPN22 protein in both CD4 and CD8 T cells. The variant allele PTPN22^R619W^ is also defective in regulating lymphopenia-induced proliferation and CD8 T cells expressing this variant also have increased production of inflammatory cytokines indicating that this mutation could pose a risk factor for the development of secondary autoimmunity in man as a sequela to Ab-mediated lymphodepletion that failed to spare Tregs.

## Discussion

In this study we tested the hypothesis that a gene, *PTPN22*, identified as a risk factor for autoimmunity might increase susceptibility to development of autoimmune conditions following lymphodepletion. Using anti-CD4 and anti-CD8 antibodies in combination we found that severe T cell depletion was followed by transient proliferation and activation of T cells until T cell homeostasis was restored. PTPN22 deficient T cells showed higher expression levels of activation markers, such as CD44, and produced more pro-inflammatory cytokines compared to WT T cells. This activated phenotype persisted amongst PTPN22 KO T cells after restoration of homeostasis but did not lead to weight loss nor the induction of colitis. Unexpectedly PTPN22 deficiency had no impact on the rate of proliferation nor on absolute lymphocyte numbers recovered after Ab-mediated depletion, provided IL-7 cytokine was available. IL-7R expression is down-regulated in response of TCR mediated signaling (20). Thus, IL-7 signaling in lymphopenic conditions is important for proliferation and survival when pMHC signals are limited, but dispensable in conditions of excessive pMHC resources (20, 21). In line with this, blockade of IL-7R revealed that TCR-mediated signals alone would drive greater expansion of PTPN22 KO T cells, as we have shown previously for OT-1 TCR transgenic cells (12).

The most striking differences we found between the two genotypes were increased levels of Tregs and IL-10 producing T cells in PTPN22 KO compared to WT mice from 2 weeks post depletion. IL-10 was shown previously to be critical for Treg control of LIP (22), and in a study by Winstead et al. Tregs were shown to be important to control colitis in response to lymphopenia (23). Tregs were more abundant in absolute number and in relative frequency within the CD4 T cell pool in PTPN22 KO mice 8 weeks post-depletion. Previous studies in PTPN22 KO mice have shown elevated Treg levels in steady state and found that PTPN22 also regulates Treg function (17, 19). By generating mixed bone marrow chimeric we showed that Tregs expanded more within the PTPN22 KO population indicating that Treg differentiation is unaffected by the presence of other PTPN22 KO hematopoietic cells and is, therefore, a cell intrinsic effect. Experiments comparing the conversion of PTPN22 WT and KO CD4 T cells into Foxp3 expressing regulatory cells suggested that PTPN22 mediated regulation of TCR signal strength is important for Treg differentiation *in vitro* and *in vivo* 45 days after transfer into Rag-1 KO recipient mice (24). Together these data suggest that the increase in PTPN22 KO Tregs comes primarily from peripheral expansion rather than *de novo* thymic output or enhanced iTreg generation.

Regulatory T cells have been shown to be important to keep conventional T cells in check and prevent further autoimmunological complications in lymphopenic conditions. Suppression of antigen mediated T cell proliferation by Tregs through CTLA-4 mediated downregulation of costimulatory CD80/CD86 expression on APCs and thus modulation of CD28 signaling is well-described (25, 26). The transfer of CTLA-4 deficient Tregs into Rag1 KO lymphopenic hosts prior to conventional CD4 infusion impairs T cell proliferation partially suggesting that other CTLA-4-independent mechanisms are involved (27). Alemtuzumab treatment in man (28) or in a humanized mouse model (29), depleted Foxp3+ Tregs less efficiently than conventional CD4 T cells similar to anti-CD4 antibody treatment in our experiments. We found that by 2 weeks post depletion the relative frequency of CD25+Foxp3+ expressing Tregs within the CD4 T cell pool was similar to the ratio of Tregs to conventional T cells under steady state. So far little is known about how lymphopenia may alter immune regulatory mechanisms such as the Treg compartment and how the reconstitution of regulatory T cells is regulated after irradiation and/or Ab-mediated depletion. A recent study of Delmarre et al. showed that autologous HSCT results in a diversification of the TCR repertoire suggesting that Tregs are functionally renewed by HSCT which might contribute to clinical improvement (30). Moreover, the success of autologous HSCT as a treatment for juvenile idiopathic arthritis has been shown to correlate with increased Treg frequency together with reprogramming of autoreactive T cells (31). In contrast, Jones et al. found that Treg suppressive capacity is improved 3–4 years after Alemtuzumab treatment in MS patients whether or not they developed secondary autoimmune disease, suggesting Treg abundance is not a determining factor in this instance (7, 28).

Given that PTPN22 is a major risk allele for autoimmunity and its role in the negative regulation of T cell activation in response to antigens with a weak TCR affinity, such as self-antigens, we hypothesized that functional PTPN22 might be critical for “safe” T cell restoration after a lymphopenic insult. The association of the PTPN22 R620W SNP with various autoimmune diseases suggests a regulatory role of functional PTPN22 for T cell activation in human. In mouse the R619W point mutation in the *Ptpn22* gene resembles the full knock-out suggesting that R619W is a loss of function mutation (32, 33). Interestingly neither the PTPN22 R619W nor the full PTPN22 KO mouse show any signs of autoinflammation or spontaneous development of autoimmunity in steady state on the C57Bl/6 background, despite the increased numbers of lymphocytes and their activated phenotype (17, 32). This may be a consequence of the relative resistance of the C57Bl/6 background to the development of autoimmunity as the 129 mouse background, in contrast, permitted the spontaneous development of disease in PTPN22^R619W^ knock-in mice (33).

Higher TCR affinity might lead to chronic signaling in PTPN22 KO T cells, which could cause the upregulation of co-inhibitory receptors and thereby limit excessive proliferation in absence of PTPN22. The co-inhibitory receptor PD-1 is known to be upregulated in response to T cell activation and negatively regulates T cell function. Transgenic male antigen specific PD-1 KO CD4 T cells outnumbered WT cells when adoptively transferred into female Rag1 KO, suggesting that PD-1 regulates tonic pMHC signaling and LIP (21). In contrast to PTPN22, PD-1 controlled proliferation but had no impact on IFNγ production indicating that negative regulation by PD-1 differs from PTPN22 and that not all co-factors have equal ability to cause autoimmunity. Notably the study of Jones et al. showed that CD4 and CD8 cell reconstitution was similar between patients with and without secondary autoimmunity, suggesting that the rate of T cell reconstitution and T cell number itself may not be a useful measurement to predict outcome in Alemtuzumab treated MS patients (7).

Based on our findings we conclude that antibody mediated T cell lymphopenia does not trigger overt auto-aggression in PTPN22 KO mice although PTPN22 deficient T cells showed a pronounced effector phenotype. High abundance of Tregs and IL-10 producing cells in PTPN22 KO mice have been previously associated with increased tolerance in a model of pancreatic islet transplantation and might suppress excessive T cell proliferation and keep activated T cell clones in check (34). T cell numbers were significantly higher in PTPN22 KO than in WT mice when IL-7 signaling was blocked indicating that the availability of IL-7 can mask dysregulated self-peptide MHC/TCR mediated proliferation during lymphopenia. IL-7 could be an important factor for T cell expansion in a TCR-signaling independent manner which might promote the expansion of a more diverse repertoire. Although the contribution and the exact mechanism of suppression by Tregs in lymphopenia induced proliferation is unclear, our results suggest that sparing regulatory T cell in therapies using lymphocytes depleting antibodies might be an efficient strategy to prevent lymphopenia mediated autoinflammatory reactions. The observation that PTPN22^R619W^ mice had a similar expansion of Tregs to the PTPN22-KO mice, raises the intriguing possibility that in man, carrying the PTPN22^R620W^ allele might even be protective against secondary autoimmunity following therapeutic Ab depletion, rather than being an increased risk factor as we originally hypothesized. Conversely, enhanced T cell activation mediated by LIP in absence of PTPN22 could promote tumor immunity and be attractive for cancer therapies.

## Data Availability Statement

The datasets generated for this study are available on request to the corresponding author.

## Ethics Statement

The animal study was reviewed and approved by UK Home Office and performed in accordance with the institutional and ethical guidelines of the University of Edinburgh.

## Author Contributions

JK designed and performed experiments, analyzed data, and wrote the manuscript. DW designed the PTPN22 R619W mutant mouse strain. AC provided intellectual input into the project and design of the PTPN22 R619W mouse. BM designed the OST-tagged *Ptpn22* gene construct and provided OST-tagged PTPN22 mouse. RZ led the overall project, designed experiments, and wrote the manuscript.

### Conflict of Interest

The authors declare that the research was conducted in the absence of any commercial or financial relationships that could be construed as a potential conflict of interest.

## Abbreviations

Ab: antibody
IFN: interferon
IL: interleukin
KO: knock-out
LIP: lymphopenia induced proliferation
mAb: monoclonal antibody
MHC: major histocompatibility complex
p: peptide
p. i.: post injection
PTPN: protein tyrosine phosphatase non-receptor
SNP: single nucleotide polymorphism
TCR: T cell receptor
Treg: regulatory T cell.
