# Supplementary figures and images for: PTPN22 Acts in a Cell Intrinsic Manner to Restrict the Proliferation and Differentiation of T Cells Following Antibody Lymphodepletion
Source: Front Immunol. 2020 Jan 28;11:52. doi: 10.3389/fimmu.2020.00052 (PMC6997546; doi:10.3389/fimmu.2020.00052)

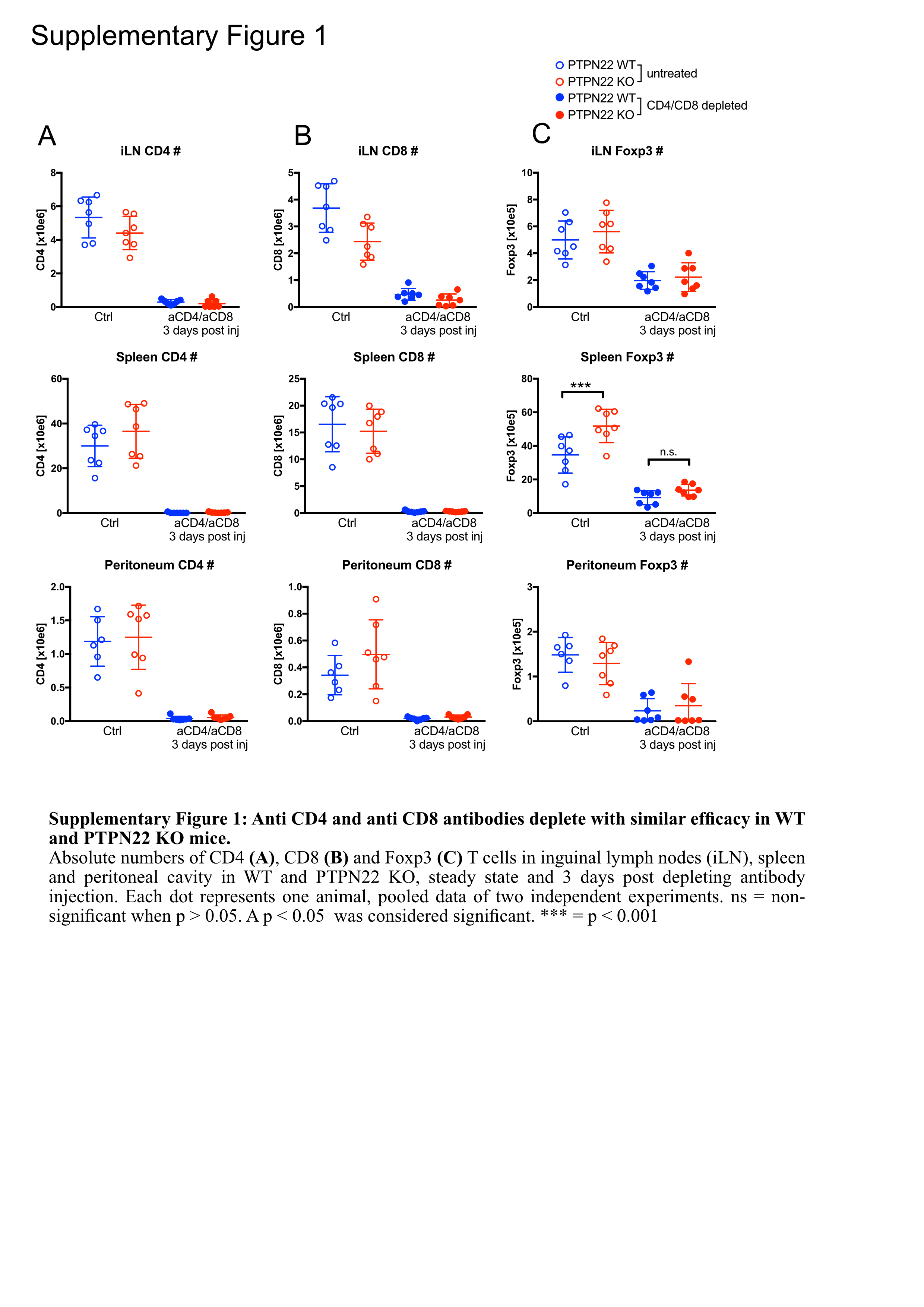

Supplement: Supplementary file 1 [file Image_1.JPEG]

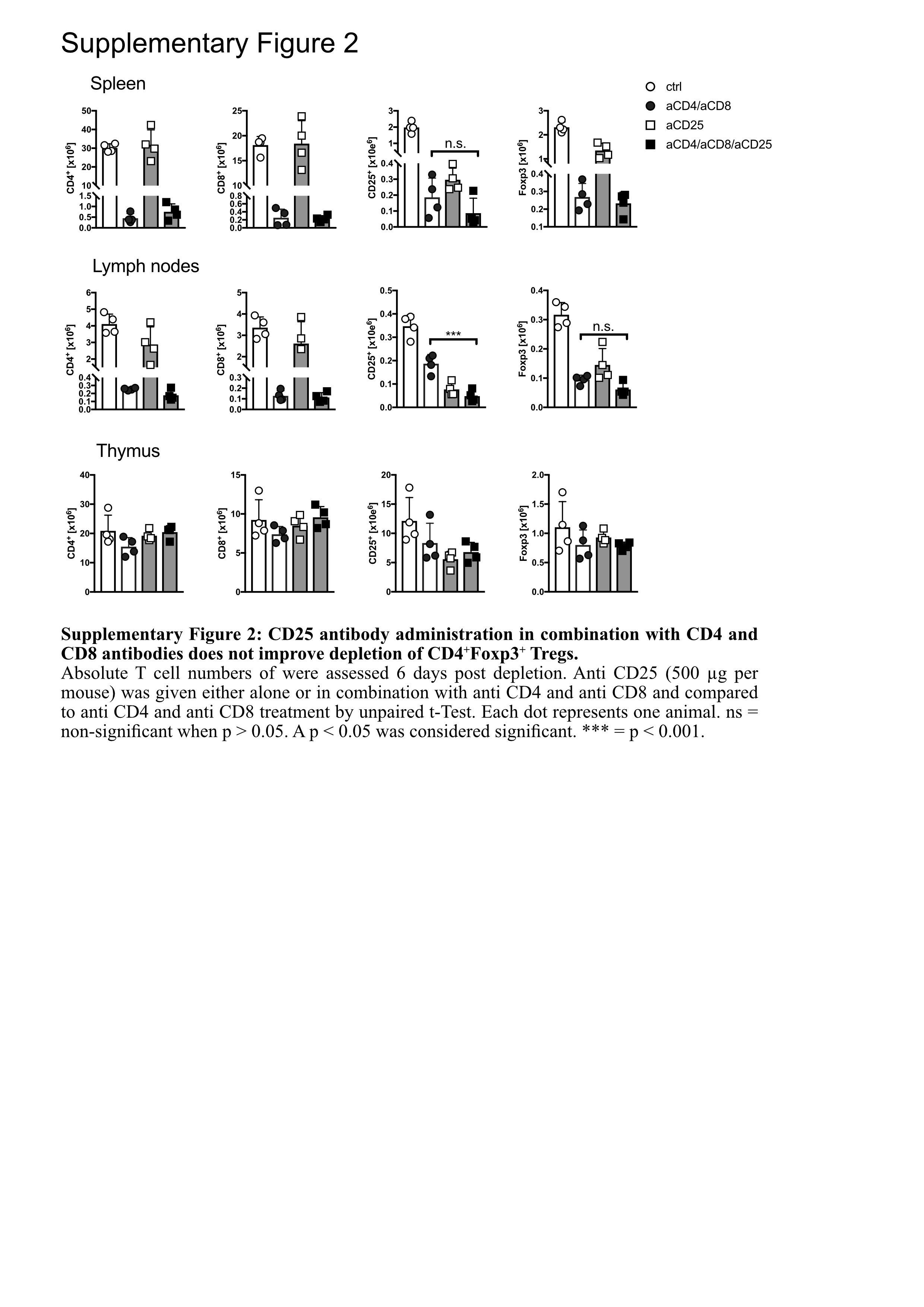

Supplement: Supplementary file 2 [file Image_2.JPEG]
